# 18F-Fluciclovine PET metabolic imaging reveals prostate cancer tumour heterogeneity associated with disease resistance to androgen deprivation therapy

**DOI:** 10.1186/s13550-020-00728-9

**Published:** 2020-11-25

**Authors:** Gaurav Malviya, Rachana Patel, Mark Salji, Rafael S. Martinez, Peter Repiscak, Ernest Mui, Susan Champion, Agata Mrowinska, Emma Johnson, Maha AlRasheedi, Sally Pimlott, David Lewis, Hing Y. Leung

**Affiliations:** 1 Cancer Research UK Beatson Institute, Garscube Estate, Switchback Road, Glasgow, G61 1BD UK; 2 Institute of Cancer Sciences, University of Glasgow, Glasgow, UK; 3 Department of Urology, NHS Greater Glasgow and Clyde, Glasgow, UK; 4 West of Scotland PET Centre, NHS Greater Glasgow and Clyde, Glasgow, UK

**Keywords:** Castration-resistant prostate cancer, Metabolic imaging, ^18^F-Fluciclovine (FACBC), Amino acid transporter, Tumour heterogeneity

## Abstract

**Background:**

Prostate cancer is highly prevalent worldwide. Androgen deprivation therapy (ADT) remains the treatment of choice for incurable prostate cancer, but majority of patients develop disease recurrence following ADT. There is therefore an urgent need for early detection of treatment resistance.

**Methods:**

Isogenic androgen-responsive (CWR22Res) and castration-resistant (22Rv1) human prostate cancer cells were implanted into the anterior lobes of the prostate in CD-1 Nu mice to generate prostate orthografts. Castrated mice bearing CWR22Res and 22Rv1 orthografts mimic clinical prostate cancer following acute and chronic ADT, respectively. ^18^F-Fluciclovine (1-amino-3-fluorocyclobutane-1-carboxylic acid) with a radiochemical purity of > 99% was produced on a FASTlab synthesiser. Ki67 staining in endpoint orthografts was studied. Western blot, quantitative RT-PCR and next-generation sequencing transcriptomic analyses were performed to assess the expression levels of amino acid transporters (including LAT1 and ASCT2, which have been implicated for Fluciclovine uptake). Longitudinal metabolic imaging with ^18^F-Fluciclovine-based positron emission tomography (PET) was performed to study tumour response following acute and chronic ADT.

**Results:**

Both immunohistochemistry analysis of endpoint prostate tumours and longitudinal ^18^F-Fluciclovine imaging revealed tumour heterogeneity, particularly following ADT, with in vivo ^18^F-Fluciclovine uptake correlating to viable cancer cells in both androgen-proficient and castrated environment. Highlighting tumour subpopulation following ADT, both SUVpeak and coefficient of variation (CoV) values of ^18^F-Fluciclovine uptake are consistent with tumour heterogeneity revealed by immunohistochemistry. We studied the expression of amino acid transporters (AATs) for ^18^F-Fluciclovine, namely LAT1 (*SLC7A5* and *SLC3A2*) and ASCT2 (*SLC1A5*). SLC7A5 and SLC3A2 were expressed at relatively high levels in 22Rv1 castration-resistant orthografts following chronic ADT (modelling clinical castration-resistant disease), while SLC1A5 was preferentially expression in CWR22Res tumours following acute ADT. Additional AATs such as SLC43A2 (LAT4) were shown to be upregulated following chronic ADT by transcriptomic analysis; their role in Fluciclovine uptake warrants investigation.

**Conclusion:**

We studied in vivo ^18^F-Fluciclovine uptake in human prostate cancer orthograft models following acute and chronic ADT. ^18^F-Fluciclovine uptakes highlight tumour heterogeneity that may explain castration resistance and can be exploited as a clinical biomarker.

## Introduction

Prostate cancer is a leading cause of premature male cancer-related death [1]*.* Despite the common practice of testing for serum prostate-specific antigen (PSA) levels in asymptomatic men, a significant proportion of patients present with advanced and/or metastatic disease. While androgen deprivation therapy (ADT) with analogue or antagonist of luteinising hormone-releasing hormone is the standard of care systemic treatment for incurable disease, increasingly docetaxel chemotherapy or additional inhibitor of the androgen receptor pathway is used in combination with standard of care ADT [2–4]. Following ADT, the majority of patients will show an initial favourable response with a dramatic fall in serum PSA levels, but will subsequently develop cancer recurrence, which is referred to as castration-resistant prostate cancer (CRPC), and remains incurable.

Metabolic imaging such as positron emission tomography (PET) has the potential to detect occult and/or persistent disease following treatment. The personalised information obtained can guide management decisions as well as support the design of future trials focussing on prostate cancer patients with the earliest signs of tumour resistance to ADT, prior to clinical evidence of cancer recurrence judged by PSA testing. Active transport of synthetic L-leucine analogues such as ^18^F labelled 1-amino-3-fluorocyclobutane-1-carboxylic acid (or ^18^F-Fluciclovine or ^18^F-FACBC) into cells is thought to be mediated by L-type neutral amino acid transporter 1 (LAT1) and alanine-serine-cysteine-transporter (ASCT2) [5, 6]*.* LAT1 is a sodium independent exchange amino acid transporter (AAT) between intracellular and extracellular environments [7]*.* In contrast, ASCT2 mediates amino acid uptake in a sodium-dependent manner [7, 8]. As a leucine analogue, ^18^F-Fluciclovine is not incorporated into proteins, but its uptake mirrors that of glutamine [6, 9], which plays a key role in cancer metabolism [7]*.* As both LAT1 and ASCT2 have been detected in prostate cancer cells, ^18^F-Fluciclovine is a promising PET radiotracer for functional prostate cancer imaging. ^18^F-Fluciclovine is already approved by the European Medicines Agency in Europe [10] and the Food and Drug Administration in the USA [11] for diagnostic imaging in prostate cancer sufferers with biochemical evidence of cancer recurrence. Favourable cancer detection performance for ^18^F-Fluciclovine PET imaging has been reported across a wide range of PSA values [12, 13].

Androgen receptor contributes to prostate carcinogenesis and treatment resistance through its effects on cancer metabolism. To date, studies on castration-resistant prostate cancer have primarily focussed on in vitro cell models. Here, we developed the CWR22Res and 22Rv1 isogenic human prostate orthografts (orthotopic xenografts) as models of androgen-responsive (dependent) and castration-resistant prostate cancer. We hypothesise that castration-resistant prostate cancer has a distinct profile of AATs expression that alters the tumoral ^18^F-Fluciclovine uptake. We applied acute and chronic ADT (in CWR22Res and 22Rv1 orthografts, respectively) as tools to mimic early and established castration-resistant disease and performed longitudinal (matched) ^18^F-Fluciclovine PET/MRI scans.

## Material and methods

### In vivo model of human prostate cancer orthografts

Isogenic androgen-responsive CWR22Res and castration-resistant 22Rv1 human prostate cancer cells were implanted into the anterior lobes of the prostate in CD-1 Nu mice to generate prostate orthografts (*n* = 4 for CWR22Res and *n* = 3 for 22Rv1) as described in Patel et al.[14]*.*

**Fig. 1:**
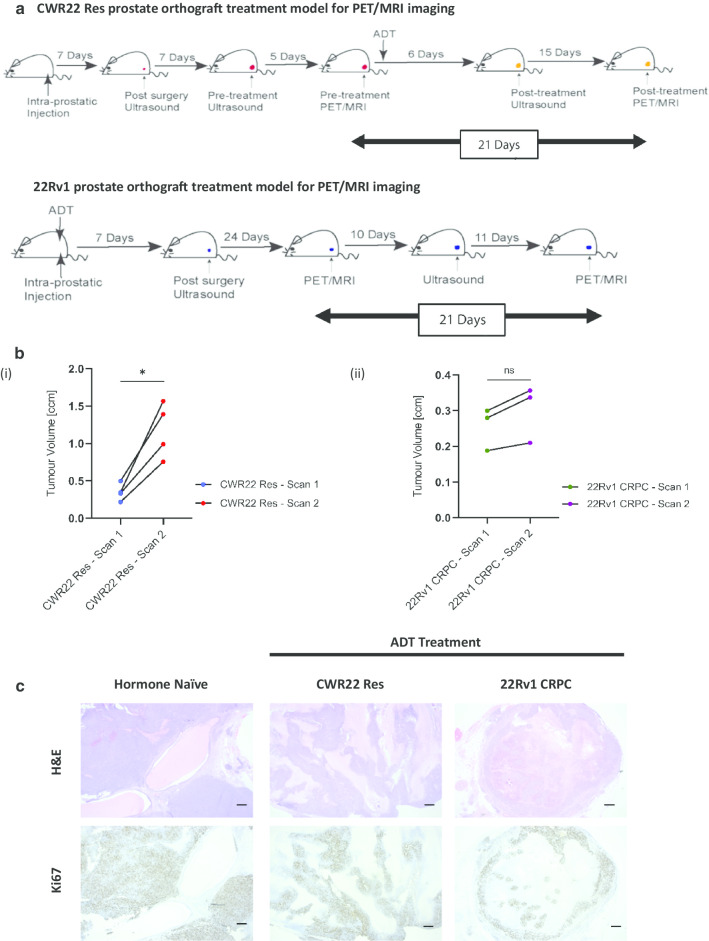
**a** Schematic illustration of in vivo prostate orthograft models (CWR22Res and 22Rv1) to mimic clinical androgen-dependent and castration-resistant prostate cancer, respectively. There was a 21-day interval between the first and second PET/MRI scans (scan 1 and scan 2) on mice bearing CWR22Res (top panel) and 22Rv1 (bottom panel) orthografts, thus recapitulating tumour responses to clinical acute and chronic ADT. **b** Comparison of tumour volumes based on data from MRI scans (i) CWR22Res Scan 1 (pre-ADT) versus CWR22Res Scan 2 (post-ADT) (*n* = 4) (paired *t* test, *p* = 0.01) and (ii) 22Rv1 under chronic ADT scan 1 *versus* scan 2 (*n* = 3) (paired *t* test, *p* = 0.06). **c** Representative images of histological analysis of orthografts at the conclusion of experiment. Androgen-dependent (hormone naïve) CWR22Res tumours were from additional control mice that were not castrated. Within the design of the experiment, there were no matched tumours for 22Rv1 at the time of the first scan (scale bar = 100 µm)

Mice bearing CWR22Res orthografts maintained in an androgen-proficient state (i.e. without castration) are referred to as hormone-naïve tumours. Two experimental groups were designed to recapitulate clinical acute ADT and chronic ADT. For acute ADT, mice bearing CWR22Res orthografts were castrated, and PET/MRI scans were performed before and after ADT (Fig. 1a top). For chronic ADT, castration-resistant 22Rv1 cells were orthotopically implanted and the mice castrated at the same time. Tumour formation was confirmed by ultrasonography, followed by two serial PET/MRI scans (Fig. 1a bottom). The effect of ADT was anticipated within three weeks, so for both acute (CWR22Res) and chronic (22Rv1) ADT treatment groups, the interval between PET/MRI scan 1 and scan 2 was 21 days.

Mice were anaesthetised using isoflurane during imaging procedures. Experiments involving the use of mice were conducted in accordance with the UK Home Office regulations (UK Animals (Scientific Procedures) Act 1986) under the Project Licence P5EE22AEE.

Sections from orthografts were prepared and analysed by haematoxylin and eosin staining as well as by immunohistochemistry for the levels of Ki67 (a marker for cell proliferation) as described in Patel et al. [14].

### ^18^F-Fluciclovine synthesis

^18^F-Fluciclovine was produced on a FASTlab synthesiser, using single use cassette kits provided by Blue Earth Diagnostics Limited. The ^18^F-Fluciclovine (or ^18^F- FACBC) was produced with a radioactivity yield of 49.1 ± 3.8% (*n* = 4) and the end of synthesis activity concentration of 1481 ± 190 MBq/ml. At end of synthesis the radiochemical purity was found to be 99.18 ± 0.13% (*n* = 4, Additional file 1: Figure S1), and the molar activity was found to be 1044.45 ± 638.14 GBq/µmol (*n* = 3). The final ^18^F-Fluciclovine product was formulated in citrate buffer and was terminally filtered through a 0.2-µm filter.

Radiochemical purity was measured using thin-layer chromatography. Thin-layer chromatography silica gel 60 aluminium sheets strips were eluted over 7.5 cm using a mobile phase consisting of acetonitrile:methanol:water:acetic acid at 20:5:5:1 v/v.

Fluciclovine content was measured using ion chromatography and electrochemical detection with an amino acid waveform, a gold working electrode and a Ag/AgCl reference electrode. Analysis was performed on a Dionex AminoPac PA10 analytical column (4 × 250 mm) eluted with 150 mM NaOH.

### ^18^F-Fluciclovine PET/MRI imaging

^18^F-Fluciclovine positron emission tomography/magnetic resonance imaging (PET/MRI) scans on mice bearing prostate orthografts were performed using a nanoScan PET/MRI scanner (Mediso Medical Imaging Systems, Hungary). Mice were maintained under inhaled isoflurane anaesthesia (induction 5% v/v; maintenance 1.5–2.0% v/v) in the medical air during injection and PET/MRI imaging procedures periods. Each mouse received 15.9 ± 4.6 MBq of ^18^F-Fluciclovine radiotracer via an intravenous bolus injection in tail vein. Static PET acquisitions were performed for 15 min after an uptake period of 50 min. Whole-body T1-weighted Gradient Echo 3D Coronal/Sagittal MRI Sequences (slice thickness 0.5 mm, repetition time 12.057 ms, echo time 3.3 ms, flip angle 15 degrees) were used to acquire MRI scans.

Image reconstruction was performed using 3D Tera-Tomo software (Mediso Medical Imaging Systems, Hungary), and PET data were corrected for radioactivity decay, random coincidences, scatter, attenuation and dead time. The reconstructed PET scans were co-registered with MRI scans for anatomical reference.

For quantitative assessments of scans, volume-of-interest (VOI) was manually drawn around the prostate on MRI scans by visual inspection using PMOD software version 3.504 (PMOD Technologies Ltd., Switzerland), and same VOI copied on to the respective PET scans. Separate VOIs were drawn for each scan to adjust for the position of the mice on the scanner and tumour size. Standardised uptake values (SUV) were determined by dividing the radiotracer concentration in VOI by the injected dose divided by the animal weight. SUVmean values were calculated using mean of all pixel values within tumour VOI. SUVmax values were derived from the hottest VOI pixel values, while SUVpeak values were calculated using mean of five hottest VOI pixel values. Coefficient of variation (CoV) was determined by dividing the standard deviation by the mean ^18^F-Fluciclovine uptake. Data were analysed using a 2-way ANOVA followed by post hoc Sidak’s multiple comparison tests in the GraphPad Prism software version 8.4.3 (GraphPad software LLC, USA).

### Western blot analysis

Cell lysates from prostate orthografts were prepared as previously described in Patel et al. [15] and Rushworth et al. [16]. Lysates were resolved by SDS-PAGE in 4–12% gradient Bis–Tris gels (Life Technologies) before wet transfer to PVDF membrane (Millipore) using the NuPage transfer module (Life Technologies). Membranes were blocked with 5% milk before incubation with primary antibody overnight at 4 °C, with anti-SLC7a5 antibody from Cell Signalling (5347S), anti-SLC3a2 antibody from Sigma-Aldrich (SAB1400263), anti-SLC1a5 antibody from Invitrogen (PA5-50,527) and anti-HSC70 antibody from Santa Cruz (sc-7298). Following incubation with secondary antibodies, using Alexa Fluor 680 goat anti-rabbit (Life Technologies) or goat anti-mouse DyLight 800 (ThermoFisher Scientific), western blot membranes were scanned using the LI-COR Odyssey CLx Imaging system (LI-COR Biosciences) and visualised with the Image Studio Lite Ver 5.2 software.

### Transcriptomic analysis of castration-resistant prostate cancer orthografts

mRNA was extracted from orthografts using RLT buffer supplemented with 1% β-mercaptoethanol according to the manufacturer’s instructions for the RNeasy Mini Kit (Qiagen). RNA was quantified using the NanoDrop 2000 spectrophotometer (Thermo Scientific). For RNA sequencing samples, RNA quality was assessed on a 2100 Bioanalyser (Agilent). Libraries from RNA samples were prepared for sequencing using the Illumina TotalPrep RNA Amplification Kit (Ambion, Life Technologies) with cytoplasmic and mitochondrial ribo-depletion (Illumina Ribo-Zero Gold Stranded Kit) according to the manufacturer’s instructions. Quality checks and trimming of the raw RNA-Seq data files were conducted using FastQC (v0.11.8), Trim Galore (v0.6.4) and FastQ Screen (v0.13.0). RNA-Seq paired-end reads were aligned to the human genome (GRCh38 v95) using HiSat2 (v2.1.0), and gene expression levels were determined using featureCounts (v1.6.4). Differential gene expression analysis, based on the negative binomial distribution, was performed using DESeq2 package (v1.26.0), and expression of significantly (*p*-adj < 0.05) differentially expressed amino acid transporters was plotted as a heatmap using pheatmap (v1.0.12). Analysis was performed in the R environment (v3.6.3).

## Results

### CWR22Res and 22Rv1 orthograft models mimic clinical hormone-dependent and castration-resistant prostate cancer

CWR22Res (hormone-dependent) and 22Rv1 (castration-resistant) human isogenic prostate cancer cells were implanted orthotopically in immune-compromised nude mice to generate orthografts. The experimental plan as outlined in Fig. 1a was designed to support longitudinal imaging analyses of in vivo tumour growth following both acute and chronic ADT in mice bearing CWR22Res and 22Rv1 orthografts, respectively.

Based on MRI imaging performed at the same time as PET, we calculated the orthograft size in each mouse at two time points 21 days apart (Fig. 1b). CWR22Res tumours continued to increase in size (*p* = 0.01, paired t test) after acute ADT (by castration). In contrast, we only observed a marginal increase in 22Rv1 tumour size (*p* = 0.06, paired *t* test), after chronic ADT (by castration), as often observed in clinical castration-resistant tumours.

We analysed the orthografts by haematoxylin and eosin staining as well as by immunohistochemistry for the levels of Ki67 (a marker for cell proliferation) (Fig. 1c). We observed areas of viable tumour positive for Ki67 expression and patchy areas of necrotic tissue stained negative for Ki67. Comparing CWR22Res orthografts before and after castration, the level of necrosis appeared higher following castration.

### ^18^F-Fluciclovine PET/MRI imaging

We performed longitudinal PET/MRI metabolic imaging on prostate orthograft bearing mice at two time points to monitor changes in ^18^F-Fluciclovine uptake following acute and chronic ADT, respectively. Mice bearing CWR22Res orthografts underwent ^18^F-Fluciclovine PET/MRI imaging prior to castration, and a follow-up PET/MRI performed at 21 days later, mimicking acute ADT. Similarly, once 22Rv1 castration-resistant prostate orthografts were confirmed in a castrated environment by ultrasonography, a ^18^F-Fluciclovine PET/MRI scan was performed, with a follow-up scan at 21 days later, recapitulating clinical chronic ADT.

**Fig. 2:**
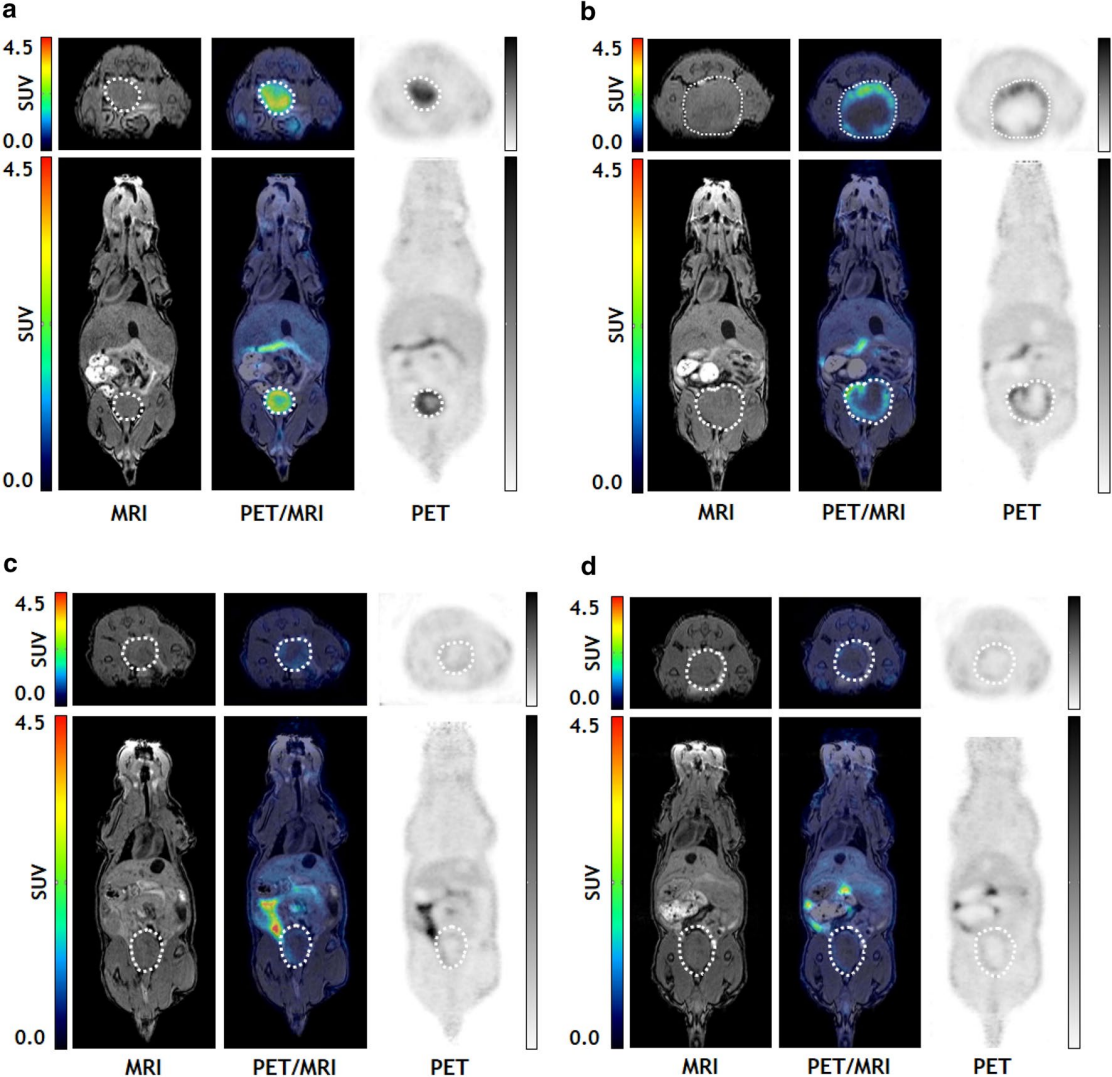
Serial (longitudinal) ^18^F-Fluciclovine PET/MRI imaging on the same mouse bearing either CWR22Res orthograft [**a** Scan 1 (before ADT) and **b** Scan 2 (after ADT)] or 22Rv1 orthograft [**c** Scan 1 and **d** Scan 2 (both following ADT)] (representative image of *n* = 4 mice for CWR22Res and *n* = 3 mice for 22Rv1 bearing mice). Images presented in axial and sagittal imaged field of view (upper panel and lower panel, respectively)

**Fig. 3:**
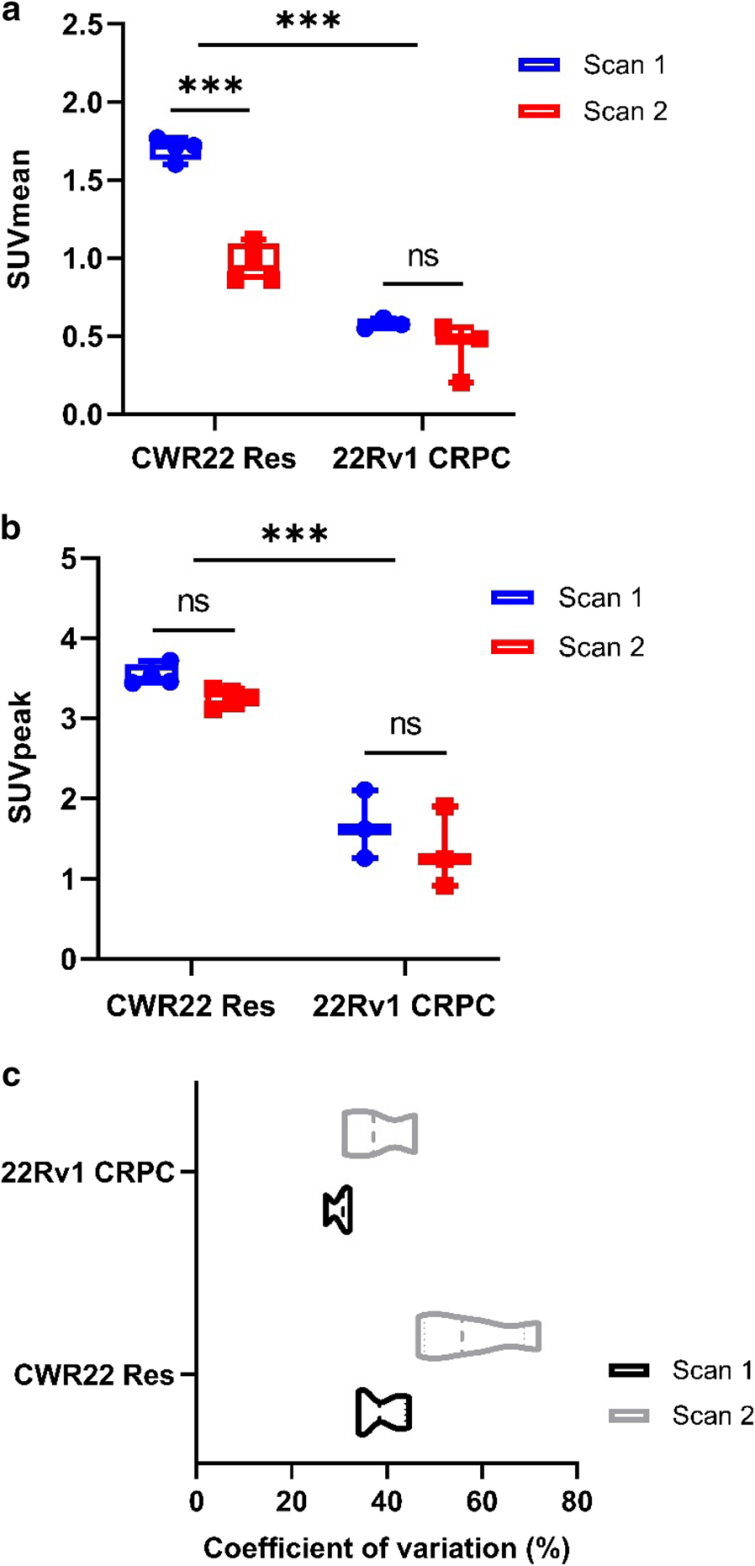
Analysis of ^18^F-Fluciclovine PET SUV values [SUVmean (**a**), SUVpeak (**b**)] of CWR22Res (hormone-dependent) and 22Rv1 (castration-resistant) orthografts (two-way ANOVA, ***p < 0.0002, ns = not significant). **c** Coefficient of variation was calculated to assess the heterogeneity of ^18^F-Fluciclovine uptake within the CWR22Res and 22Rv1 orthografts. (*n* = 4 for CWR22Res and *n* = 3 for 22Rv1 orthografts)

Analysis of matched SUVmean values for CWR22Res orthografts before and after acute ADT identified a significant reduction in SUVmean (*p*-adj = 0.0002; two-way ANOVA), signifying a global reduction of tumoral ^18^F-Fluciclovine uptake following castration (Fig. 2a, b, 3a). In contrast, the castration-resistant 22Rv1 orthografts maintained SUVmean values between the two scans (*p*-adj = 0.1428; two-way ANOVA), albeit at relatively low levels (Fig. 2c, d, 3a), suggesting sustained amino acid transporter expression during in vivo CRPC growth under chronic ADT condition. There was consistently less ^18^F-Fluciclovine uptake in castrate-resistant 22Rv1 tumours compared to hormone-sensitive CWR22Res orthografts (*p* < 0.0001; two-way ANOVA).

Tumour heterogeneity and plasticity represent an important cellular adaptive response to overcome selective pressure from castration. Consistent with findings from histopathology analysis (Fig. 1c), tumour heterogeneity was evident on PET/MRI imaging (Fig. 2). We therefore carried out comprehensive SUV analyses to include data from SUVmean, SUVpeak, SUVmax and coefficient of variation on PET image data (Fig. 3, Additional file 1: S2). Despite significant change in the SUVmean for CWR22Res orthografts following castration (Fig. 3b), the respective SUVpeak and SUVmax values before and after castration were comparable (Fig. 3c, Additional file 1: S2). For 22Rv1 orthografts, SUVmean, SUVpeak and SUVmax values were all sustained in the longitudinal scans. Consequently, tumour heterogeneity, as measured by the coefficient of variation across the tumour volume, increased in acute ADT (*p*-adj = 0.0099, two-way ANOVA) but not chronic ADT (*p*-adj = 0.2513) (Fig. 3c). Collectively, our SUV analyses support the concept that a tumour subpopulation with sustained ^18^F-Fluciclovine uptake following castration may represent cancer resistant to ADT and may therefore contribute to the development of CRPC.

### Analysis of amino acid transporters (AATs) expression

**Fig. 4:**
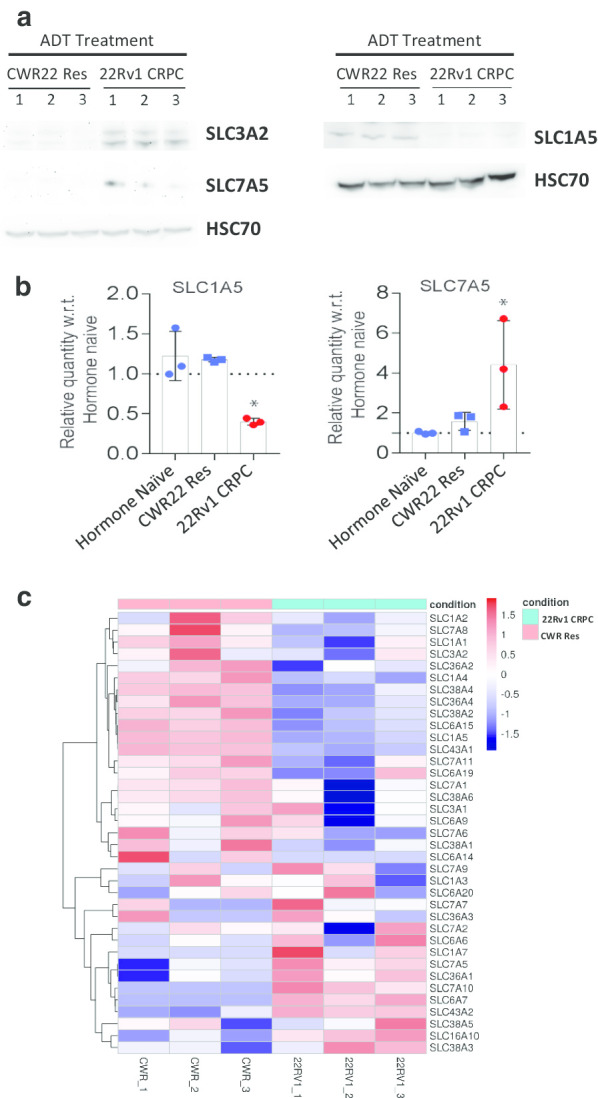
**a** Western blot analysis of lysates from endpoint CWR22Res and 22Rv1 orthografts in castrated mice. Each lane contains lysate from an individual tumour. HSC70 used as loading control. (Molecular weights: SLC3A2, 80–85 kDa; SLC7A, kDa, 39 kDa; SLC1A5, 56 kDa; HSC70 70 kDa) (Left and right panels were each performed on a single blot respectively). **b** Quantitative RT-PCR analysis of SLC1A5 and SLC7A5 mRNA expression in orthografts. Hormone-naïve CWR22Res tumours obtained from uncastrated (androgen-proficient) mice were included as controls. CWR22Res and 22Rv1 tumours from experimental castrated mice mimic clinical tumours following acute and chronic ADT (*n* = 4 for CWR22Res, *n* = 3 for 22Rv1). Each data point is data from an individual tumour, with six technical repeats for each sample. **c** Heatmap showing mRNA expression levels (*z* scores) of significantly (*p*-adj < 0.05) differentially expressed amino acid transporters. Each sample analysed was from an individual tumour from endpoint mice bearing CWR22Res and 22Rv1 orthografts

^18^F-Fluciclovine (or ^18^F-FACBC) is a synthetic L-leucine analogue taken up by cells via amino acid transporters (AATs), with the LAT1 and ASCT2 transporters particularly implicated [5]*.* We assayed the expression levels of relevant amino acid transporters at both protein and mRNA levels. LAT1 consists of a heterodimer from the gene products of *SLC7A5* and *SLC3A2*, while ASCT2 is encoded by *SLC1A5*. On western blotting, SLC7A5 and SLC3A2 were both upregulated in 22Rv1 orthografts, when compared to CWR22Res orthografts following castration (Fig. 4a). In contrast, 22Rv1 castration-resistant orthografts expressed SLC1A5 at a lower level than the androgen-dependent CWR22Res orthografts (Fig. 4a). We further performed quantitative RT-PCR to assay for mRNA levels of *SLC1A5* (or ASCT2) and *SLC7A5* (part of LAT1) and confirmed reduced ASCT2 and increased LAT1 expression in 22Rv CRPC compared to CWR22Res androgen-dependent tumours (Fig. 4b).

To further explore potential AAT targets for ^18^F-Fluciclovine uptake in our in vivo treatment-resistant prostate cancer model, we utilised data from transcriptomic analysis of prostate orthografts [17]*.* Expression levels of significantly (*p*-adj < 0.05) differentially expressed AAT are presented as a heatmap (Fig. 4c). SLC7A5 and SLC1A5 mRNA expression was consistent with that observed at protein level, i.e. elevated SLC7A5 and reduced SLC1A5 levels in the castration-resistant 22Rv1 tumours. Besides sustained SLC7A5 (LAT1) mRNA expression, we observed upregulated expression of SLC43A2 and SLC6A7. SLC43A2 (Solute Carrier Family 43 Member 2 or Large Neutral Amino Acids Transporter Small Subunit 4, LAT4) is the most significantly upregulated AAT (log2FC = 1.78, *p*-adj = 1.32E-0.9) in 22Rv1 CRPC (Additional file 1: Table S1).

## Discussion

Metabolic imaging is increasingly applied in the management of cancer patients, particularly for the detection of occult and/or persistent disease following treatment. The current standard of care imaging with the use of CT, MRI and isotopic bone scan cannot detect small foci of primary and metastatic disease. PET/MRI imaging with ^18^F-Fluciclovine, a radiolabelled synthetic amino acid, in prostate cancer patients may locate disease recurrence and guide management decision in a personalised manner [18]. Unlike ^18^F-fluorodeoxyglucose, it is not absorbed by all cells with an increased glucose metabolic rate, but specifically targets cell surface amino acid transporters ASCT2 and LAT1 overexpressed in prostate cancer.

We have optimised a preclinical orthotopic xenograft (orthograft) mouse model of prostate cancer to mimic clinical castration-resistant disease and examined the application of longitudinal 18F-Fluciclovine-based imaging in treatment-resistant prostate cancer. We identified intra-tumoral heterogeneity following androgen deprivation therapy, corroborating data from immunohistochemistry analysis with PET signals. Consistent with the literature adaptive upregulation of LAT1 expression in castration-resistant tumours was observed. In addition, unbiased transcriptomic analysis implicated upregulated SLC43A2 (LAT4) expression as a candidate amino acid transporter associated with resistance to castration.

Majority of patients following androgen deprivation therapy have excellent drop in serum PSA levels. Within the current clinical practice, we are unable to identify patients at high risk of early disease recurrence. Data from longitudinal 18F-Fluciclovine PET imaging revealed intra-tumoral heterogeneity that may explain cancer subpopulation surviving androgen deprivation therapy (castration). These viable tumour cells signify the basis of subsequent recurrent tumour. In the future, 18F-Fluciclovine PET imaging following treatment may help clinicians to identify patients at high risk of early cancer recurrence and therefore provide the opportunity to consider additional treatment, while the bulk of resistant cancer remains low.

Besides ^18^F-Fluciclovine-based imaging, other PET tracers including [^11^C]choline and PSMA (prostate-specific membrane antigen)-based tracers have also been investigated following activation or suppression of the androgen receptor [19–21]. Comparing the accuracy of ^18^F-Fluciclovine and [^11^C]choline PET imaging in a cohort of 100 patients with biochemical recurrence following previous radical treatment, ^18^F-Fluciclovine had better true-positive, false-positive, true-negative and false-negative rates (Chi-squared test, *p* < 0.0001) as well as practical advantage over [^11^C]choline, including ease of handling and scan interpretation [22]. ^18^F-Fluciclovine PET also performs favourably in detecting recurrent disease, including nodal metastasis [23] (with pathologic confirmation [24]), when compared to [^11^C]choline PET.

In a recent head-to-head comparative study in patients with recurrent prostate cancer [20], while ^18^F-Fluciclovine and [^68^ Ga]Ga-PSMA-11 PET imaging had similar detection rates (79.3%, ^18^F-fluciclovine; 82.8%, [^68^ Ga]Ga-PSMA-11; p = 0.64), ^18^F-fluciclovine was more efficient in detecting local recurrence in 37.9% of examined cases (compared to 27.6% for [^68^ Ga]Ga-PSMA-11, *p* = 0.03). However, additional comparative studies are required to determine the PET tracer of choice to detect CRPC with confidence.

Orthografts generated from CWR22Res and derived 22Rv1 cells represent excellent preclinical models for androgen-dependent and castration-resistant prostate cancer. Importantly, these novel mouse models demonstrated to be relevant to clinical prostate cancer, particularly castration-resistant prostate cancer. Surgical castration has the benefit of instantaneously achieving androgen deprivation, providing a clear time point when effective hormone treatment commences. This contrasts with chemical castration which often takes six weeks to reach optimal effects. Once androgen deprivation therapy is in place, substantial effects on the prostate cancer are expected within two to three weeks. The interval of three weeks between first and second scans ensures that we detect tumour response to castration as well as demonstrate evidence of viable tumour cells that may subsequently develop into recurrence disease.

CWR22Res and 22Rv1 maintain the expression of androgen receptor. We observed generally higher levels of ^18^F-Fluciclovine uptake in androgen-proficient environment. Nevertheless, in castrated environment, ^18^F-Fluciclovine ‘hot spots’ help to highlight viable tumour cells following both acute and chronic ADT. We examined the application of longitudinal ^18^F-Fluciclovine-based imaging in treatment-resistant prostate cancer and identified tumour heterogeneity, which was supported by morphologic findings from immunohistochemistry analysis.

The levels of ^18^F-Fluciclovine uptake differ in tumours growing in androgen-proficient and castrated conditions. The thresholds and SUV parameters (namely SUVmean, SUVpeak and SUVmax) adopted for clinical ^18^F-Fluciclovine PET reporting may therefore require appropriate assessment and may vary in different (namely treatment naive or castration resistant) clinical context. Collectively, our data are consistent with the notion that viable tumour subpopulations (highlighted by SUVpeak, SUVmax and CoV) may be involved in treatment resistance and can be detected by longitudinal ^18^F-Fluciclovine imaging. Future prospective studies to follow patients receiving ADT are warranted.

The experimental design of our study enables direct comparison of data obtained from the longitudinal PET/MRI scans. It was not possible to obtain matched tumour materials from the time of the first scan to carry out molecular analyses using methodologies such as immunohistochemistry, western blotting and next-generation sequencing transcriptomic studies. Nonetheless, we were able to confirm upregulated expression of LAT1 following chronic ADT at both protein and mRNA levels, while ASCT2 (SLC1A5) expression was at low level in 22Rv1 CRPC tumours under chronic ADT.

Analysis of amino acid transporter expression in the context of Fluciclovine uptake has been studied using in vitro prostate cancer cell models. ASCT2 (SLC1A5) was shown to correlate with ^14^C-Fluciclovine uptake in androgen receptor-positive human LNCaP prostate cancer cells in vitro [25, 26]*.* In a study looking at the dynamics of ASCT2 across different prostate cancer states, ASCT2 expression appeared to have an overall minor decrease from androgen-sensitive to castration-resistant states, which is consistent with our data [27]. The ‘ying-yang’ pattern of LAT1 expression between androgen-sensitive and castration-resistant states observed in our study fits with what has been previously reported [28]. More recently, consistent with our findings, high levels of LAT1 expression in resected prostate tumours positively and significantly correlated with tumoral ^18^F-Fluciclovine uptake [8]*.* Hence, the in vivo model of CRPC presented here mimics clinical disease and further builds on previously studies based on in vitro models*.*

Finally, we presented comprehensive transcriptomic analysis of AAT expression in CRPC and identified a number of novel AATs such as LAT4 being upregulated. LAT4 is involved in sodium-, chloride- and pH-independent transport of L-isomers of neutral amino acids such as leucine, phenylalanine, valine and methionine. The role of LAT4 in the development of CRPC and in ^18^F-Fluciclovine uptake therefore warrants further investigation.

## Conclusion

Persistence of ^18^F-Fluciclovine uptake may signify the presence of tumour resistance to ADT, and upregulated tumoral expression of LAT1 following chronic ADT may at least in part mediate ^18^F-Fluciclovine uptake. Detecting castration-resistant cancer cells may facilitate patient stratification to receive additional treatment. Future clinical trials can incorporate the use of a post-treatment PET scan to identify patients suitable for adjuvant trial treatment.

## Key points

### Question

What is the in vivo relationship between androgen deprivation therapy and tumoral uptake of ^18^F-Fluciclovine PET tracer in the context of castration-resistant prostate cancer?

### Pertinent findings

Using novel isogenic prostate orthografts to model clinical prostate cancer, ^18^F-Fluciclovine uptake highlighted tumour heterogeneity and treatment resistance. LAT1 (and not ASCT2) is implicated as a potential AAT for ^18^F-Fluciclovine uptake in CRPC, and additional AAT (e.g. SLC43A2/LAT4) is implicated by unbiased transcriptomic analysis.

### Implications for patient care

^18^F-Fluciclovine PET imaging may detect CRPC at the earliest stage of tumour resistance.

## Supplementary information

**Additional file 1.** Supplementary information.

## Data Availability

Relevant data in support of this paper can be requested from the corresponding authors. Experiments involving the use of mice were conducted in accordance with the UK Home Office regulations (UK Animals (Scientific Procedures) Act 1986) under the Project Licence P5EE22AEE.

## Abbreviations

ADT: Androgen deprivation therapy
MRI: Magnetic resonance imaging
PET: Positron emission tomography
PSA: Prostate-specific antigen
CRPC: Castration-resistant prostate cancer
VOI: Volume-of-interest
SUV: Standardised uptake value
CoV: Coefficient of variation
