# Supplementary material for: 18F-Fluciclovine PET metabolic imaging reveals prostate cancer tumour heterogeneity associated with disease resistance to androgen deprivation therapy
Source: EJNMMI Res. 2020 Nov 25;10:143. doi: 10.1186/s13550-020-00728-9 (PMC7688773; doi:10.1186/s13550-020-00728-9)

**Table S1**

Significantly differentially expressed amino acid transporters (padj < 0.05) in 22RV1 vs CWR comparison. Genes are ordered based on decreasing log2FC.

**Figure S1.**

An example thin layer chromatograph taken from the analysis of final ^18^F-Fluciclovine product showing a radiochemical purity of >99%.


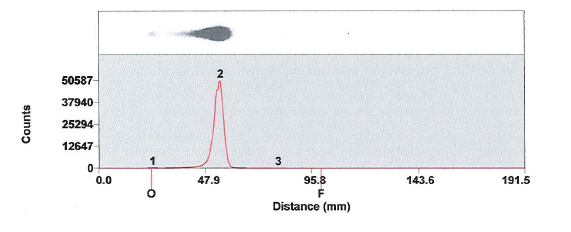


**Figure S2.**

Analysis of ^18^F-Fluciclovine PET SUVmax values of CWR22Res (hormone dependent) and 22Rv1 (castration-resistant) orthografts (two-way ANOVA, ***p < 0.0002, ns= not significant). (n=4 for CWR22Res and n=3 for 22Rv1 orthografts).


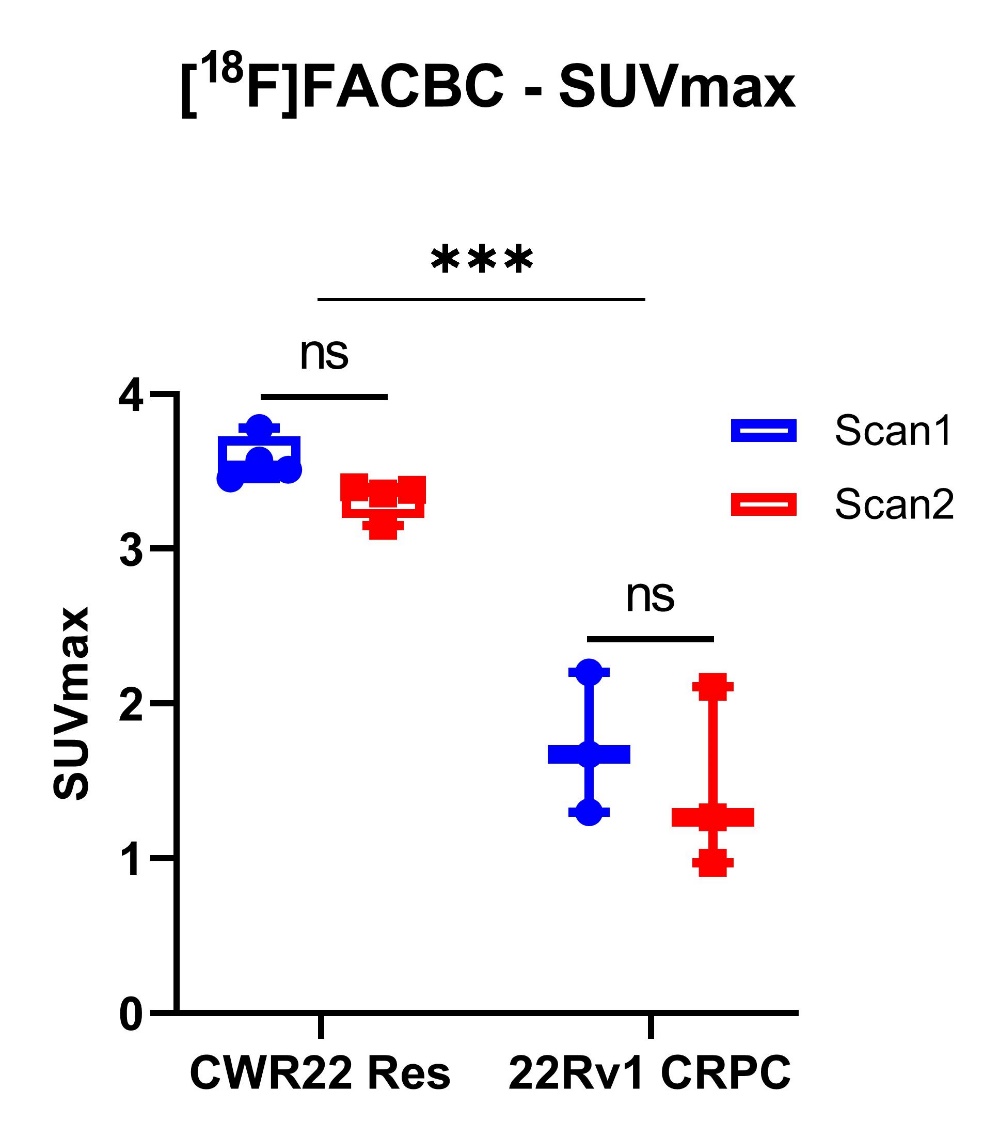

Supplement: Supplementary file 1 — Additional file 1. Supplementary information. [file 13550_2020_728_MOESM1_ESM.docx]
